#  Transition to Psychosis: Evaluation of the First-Degree Relatives of Patients with Schizophrenia ‎

**Published:** 2016-01

**Authors:** Mehdi Hormozpour, Homayoun Amini‎, Sara Pajouhanfar, Masoomeh Faghankhani, Arash Rahmani, Vandad Sharifi‎

**Affiliations:** 1 Department of Psychiatry, Tehran University of ‎Medical Sciences, Tehran, Iran; 2 Iran University of Medical Sciences, Tehran, ‎Iran; 3 Tehran University of Medical Sciences, Tehran, ‎Iran; 4 Mental Health Research Center, Tehran Institute of Psychiatry- School of Behavioral Sciences and Mental Health, Iran University of Medical Sciences, Tehran, Iran; 5 Psychiatry and Psychology Research Center, ‎Tehran University of Medical Sciences, Tehran, ‎Iran

**Keywords:** Psychosis, Schizophrenia, Prodrome, Relatives

## Abstract

**Objective: **Schizophrenia and other psychoses have devastating personal and social impacts and many efforts have been devoted to study ‎prodromal syndromes for psychosis in order to achieve earlier detection and interventions. However, only few studies have been ‎performed in developing countries on this subject, and there is a dearth of evidence in the Iranian population. In this study, we ‎focused on conversion rate to psychosis and changes in prodromal symptoms in a group of first-degree relatives of patients with ‎schizophrenia and to compare the conversion rate in those with and without prodromal symptoms as assessed by the Structured ‎Interview for Prodromal Syndromes (SIPS) and Scale of Prodromal Symptoms (SOPS).‎‎

**Method:** Participants were the first-degree relatives of hospitalized patients with schizophrenia at Roozbeh Hospital, Tehran, Iran. At baseline, ‎a trained psychiatrist interviewed the participants using the SIPS and the SOPS and assigned them to high- or low-risk groups either ‎based on the presence of prodromal criteria or seeking mental health services. After 12 months, the same examiner re-evaluated ‎the participants in order to determine the changes in their symptoms and identify the probable transitions to psychosis.‎

**Results:** One hundred participants, 50 participants within each of high- or low-risk groups, were recruited at baseline. Eight participants ‎dropped out of the study. At the follow-up, the rate of transition to full psychosis among high-risk group was 13% (95% CI [0.029, ‎‎0.23]), whereas none of the low-risk participants developed psychosis. None of the high-risk participants demonstrated attenuation ‎in their prodromal states after a one-year follow-up. In contrast, of the 50 low-risk participants, three experienced prodromal ‎symptoms for psychosis during this period. High-risk participant’s illustrated higher severity in almost all of the SOPS items compared ‎to the low-risk participants at both baseline and follow-up evaluations.‎

**Conclusion**: Prodromal syndrome for psychosis based on the SIPS and the SOPS was a predictive factor for transition to psychosis after a 12-‎month period in a group of first-degree relatives of patients with schizophrenia admitted to a psychiatric hospital in Iran. Conducting ‎further studies on this at-risk population is highly recommended in order to provide practical methods for early screening and ‎therapeutic interventions.‎

Schizophrenia and other psychoses are among the leading mental illnesses contributing to the global burden of diseases (1). ‎Schizophrenia affects approximately about seven to eight individuals per 1,000 during their lifetime worldwide (2), and imposes ‎considerable economic burden globally, including direct healthcare costs and indirect costs associated with loss of productivity (3–8). ‎Schizophrenia and other psychoses have debilitating impacts on the patients’ well-being and dramatically impair their functioning in ‎all aspects of their lives. In addition to economic burden, it also has considerable impact on relatives, caregivers and others who are ‎in contact with patients (9–12). In response to such devastating personal and social costs of schizophrenia, early identification in the ‎hopes of preventing psychosis or at least delaying it at “pre-psychosis” or prodromal period seems to be a rational choice. Although symptoms of pre-psychosis have been recognized since 1959 by Meares (9), early identification and intervention programs in the ‎pre-psychosis period dates back to the early 1990s. Among those initiating this movement are McGorry, Yung and colleagues in ‎Australia in 1994, and Miller and colleagues at Yale University, which then spread worldwide. The pre-psychosis period is the time ‎interval between the first noticeable changes in the behavior to the appearance of overt psychotic symptoms of schizophrenia and is ‎variably called “clinical high risk” (CHR), “ultra-high risk“(UHR), or “(putatively) prodromal” (13–15). ‎

The prodromal state period that on average can last days up to years for five years (16, 17) is a golden time to initiate treatment ‎towards better prognosis based on two major arguments (18). First is the reduction of the duration of untreated psychosis (DUP) as it ‎is shown that prolonged DUP has a devastating role in biological functioning of the brain (19). In addition, it is hypothesized that poor ‎prognosis of earlier illness onset may be related to a prolonged DUP rather than solely the younger age of onset. Schimmelmann ‎and colleagues have shown that reducing the duration of untreated psychosis equipoised the effect of earlier illness onset as a poor ‎prognosis factor (20). Second is the prevention of the pronounced functional decline as one of the major predictors of the transition ‎from prodromal state to full psychosis. It is also shown in retrospective studies that patients dated the onset of their functional ‎decline back to the prodromal, pre-psychotic phase (17). Therefore, early detection and initial treatment strategy of prodromal ‎phase of psychosis should become a major goal of psychiatric services in order to delay the onset of full psychosis (21), reducing ‎unnecessary suffering and increasing the possibility of improved long-term outcome (22, 23).‎

Two sets of instruments are widely employed to diagnose prodromal syndromes and measure the severity of associated symptoms. ‎First is the Comprehensive Assessment of At-Risk Mental States (CAARMS) developed by the Personal Assessment and Crisis ‎Evaluation Clinic in Melbourne, Australia, and the other is the Structured Interview for Prodromal Syndromes (SIPS) developed by the ‎Prevention through Risk Identification, Management, and Education (PRIME) prodromal research team at Yale University. The ‎severity of symptoms of SIPS criteria are measured by a comprehensive tool called the Scale of Prodromal Symptoms (SOPS) ‎developed by Miller et al. and McGlashan et al. (24, 25).‎

Previous investigations have reported the transition rates to psychosis ranged from 6.6% (26) to 54% (27) with a mean follow-up ‎range of 6 to 12 months (9). Only one study with the 9.6-year follow-up reported 70% conversion rate in patients identified by basic ‎symptoms (28). The highest likelihood of conversion was found to occur within the first year after recognition of the psychosis risk ‎syndrome with no or significantly smaller further conversion rates thereafter (9). It is confirmed by some studies that reported the ‎transition rate point by point during the follow-up (18, 29 and 30). ‎

First-degree relatives are among the best candidates for the detection of the prodromal syndromes and the implementation of any ‎early intervention and prevention efforts. To our knowledge, to date, no study has focused on the prognostic value of prodromal ‎syndromes specifically based on SIPS and SOPS in this at-risk population. Therefore, we aimed to compare transition rates to full ‎psychosis among first-degree relatives of patients with schizophrenia, with respect to their prodromal states for psychosis. In ‎addition, we compared the changes in the severity of prodromal symptoms between high-risk and low-risk groups during this period. ‎

## Materials and Method

***Study Setting & Ethical Considerations:***

The original study was approved by the Institutional Review Board at Tehran University of ‎Medical Sciences, Tehran, Iran. Participants in this study provided informed consent for ‎participating in the study, including unidentified publication of the results and follow-up for ‎further investigations. No further evaluation was performed in case of disagreements. All ‎collected data were treated in line with the ethical guidelines of the medical research, and ‎anonymity of research participants was maintained.‎

***Participants:***

We selected the participants from the first-degree relatives of patients with schizophrenia who ‎were hospitalized at Roozbeh Hospital, Tehran, Iran. These patients were diagnosed by an ‎attending psychiatrist and admitted to the hospital because of an exacerbation of their ‎illnesses. Participants had to be a first-degree relative of these patients; i.e., a biological ‎parent, a sibling, or an offspring. All family members aged 15 to 35 years were approached and ‎as many who consented were included. ‎

Participants were excluded from the study if they had a past history of any axis І disorders, a ‎history of taking antipsychotics/mood stabilizers for longer than 1 months, a past history of ‎medical conditions that could present with psychotic features, or any physical/mental ‎impairments which prevented proper communication with the interviewer.‎

Participants were classified into two groups at baseline: High- and low-risk for psychosis. The ‎high-risk group consisted of family members who were diagnosed with prodromal syndromes ‎for psychosis using the Structured Interview for Prodromal Syndromes (SIPS) or reported a ‎history of any psychiatric illnesses except psychotic disorders or a self-reported need to seek ‎mental health services. Due to time constraints, we determined the score 2 instead of 3 in APS ‎‎(Attenuated Positive Syndrome) subscale as a cutoff for diagnosis of being prodromal for ‎psychosis. The participants in the low-risk group were the family members who did not fulfill ‎any of the above criteria. Fifty participants were recruited for each group, and both groups ‎were matched for age and sex. ‎

***Structured Interview for Prodromal Syndromes (SIPS):*** ***‎***

The Structured Interview for Prodromal Syndromes (SIPS) was used to investigate prodromal ‎syndromes and measure the severity of associated symptoms. It was developed by Miller et al. ‎in New Haven, CT, USA (31) and consists of the Criteria of Prodromal Syndromes, Scale of ‎Prodromal Syndromes (SOPS), General Assessment of Functioning (GAF) (32), a checklist for ‎schizotypal personality disorder and the questionnaire of family history of mental illness. The ‎SIPS offers operative concept of three prodromal syndromes as follows: Brief Intermittent ‎Psychotic Symptom syndrome (BIPS), Attenuated Positive Symptom syndrome (APS), and ‎Genetic Risk and Deterioration syndrome (GRD). Participants with BIP should have experienced ‎one or more prodromal symptoms in the psychotic severity, with the symptom(s) having begun ‎within the past three months, and experienced them for several minutes per day at a frequency ‎of at least once per month. APS is a mild or attenuated positive syndrome in the form of ‎unusual thought content (delusional ideas, persecutory ideas, or grandiose ideas), perceptual ‎abnormalities, and disorganized speech that have appeared in the past year and experienced at ‎least once per week in the past month. In GRD participants had a significant drop in functioning ‎‎ (i.e., at least a 30% drop in the GAF scale) in the past year, and had a genetic risk in the form of ‎having a first-degree relative with any psychotic or schizotypal personality disorder (25, 31).‎

The predictive validity of SIPS criteria was examined in several other studies that selected ‎individuals with prodromal symptoms and followed them prospectively measuring naturalistic ‎conversion rates; these studies mostly used the English, Spanish, and Korean versions (33–35).‎

The severity of symptoms of SIPS criteria were measured by a comprehensive tool called the ‎Scale of Prodromal Symptoms (SOPS) developed by Miller et al. and McGlashan et al. (25, 31). ‎SOPS evaluates 5 positive symptoms, 6 negative symptoms, 4 disorganization symptoms, and 4 ‎general symptoms (Table 1).‎

***Data Collection:***

The same senior resident of psychiatry who was trained conducted the interviews using the ‎instrument. The participants were interviewed by the same interviewer at two time points: At ‎baseline and one- year follow-up. We asked the participants to come to the hospital for the in-‎person interview, but a small number of participants (N = 29) who were not able to travel to the ‎hospital, interviews were conducted via phone.‎

***Statistical Analysis:***

All data from the baseline and follow-up evaluations were entered into the Statistical Package ‎for the Social Sciences (SPSS) Software Version 20 (IBM Corp. Released 2011. IBM SPSS ‎Statistics for Windows, Version 20.0. Armonk, NY: IBM Corp). In all analytical comparisons, a ‎two-sided P-value <0.05 was defined as a statistically significant level to refuse the underlying ‎null hypothesis.‎

1. Reliability

Internal consistency was assessed by computing alpha coefficients for each of the SOPS ‎subscales and total scale scores. Cronbach’s alpha coefficients were estimated for both baseline ‎and follow-up evaluations.‎

2. Validity

Spearman correlation test was applied to evaluate criterion validity of the SOPS total score in ‎relation with GAF scores at both baseline and follow-up time points. The underlying hypothesis ‎was to assess whether the instrument is valid enough to determine the changes in SOPS with ‎respect to the changes in GAF presumed to affect SOPS. ‎

3. Description

The mean and standard deviation (SD) was used to describe numerical variables, whereas the ‎relative frequency percentage was used to describe nominal and categorical variables.‎

4. Analytical Comparisons

Our data did not show a normal distribution; therefore, non-parametric analytical assessments ‎were employed. Mann Whitney U and chi square tests were applied to compare the differences ‎between high-risk and low-risk groups.‎

## Results

***Baseline characteristics:***

Among 216 participants who were invited, 100 accepted to participate in our study. Participants were assigned into two groups: ‎High- (N = 50) and low-risk (N = 50) based on the presence of prodromal syndromes. Eight participants (4 from each group) refused ‎to participate in the follow-up investigations. No difference was found in demographic characteristics between participants who ‎completed the study and those who refused to participate in the follow-up evaluation. Basic socio-demographic and clinical data for ‎‎92 participants who completed the study are presented in Table 2. No significant difference was found between the high-risk and ‎low-risk groups regarding any of the demographic and clinical characteristics. The mean age of the participants in the high-risk ‎group was 27.5(SD 5) as well as 26.7(SD 5.2) in the low-risk group. Near half of the participants were female in both high-risk and ‎low-risk groups (47.8% vs 43.5%) and one-third of the participants were married (28.3% vs 32.6%), respectively. Similar proportions ‎of the participants (23.9%) in both groups were employed.‎

**Table 1:** Items of the Scale of Prodromal Symptoms (SOPS)

**Symptom Classification**	**Items**
Positive Symptoms	P1. Unusual thought content/Delusional ideas P2. Suspiciousness/Persecutory ideas P3. Grandiosity P4. Perceptual abnormalities/Hallucinations P5. Disorganized communication
Negative symptoms	N1. Social anhedonia or withdrawal N2. Avolition N3. Decreased expression of emotion N4. Decreased experience of emotions and self N5. Decreased ideational richness N6. Deterioration in role functioning
Disorganization symptoms	D1. Odd behavior or appearance D2. Bizarre thinking D3. Trouble with focus and attention D4. Personal hygiene/Social attentiveness
General symptoms	G1. Sleep disturbance G2. Dysphoric mood G3. Motor disturbances G4. Impaired tolerance to normal stress

**Table 2:** Baseline Demographics of the Groups with High and Low Risk for ‎Psychosis

	No. (%)			
	**Prodrome**	**Prodrome**		
	**Positive**	**Negative**		
**Characteristics**	**(N=46)**	**(N=46)**		
Age, mean(SD), yrs	27.52(4.99)	26.69(5.24)		
**Female sex**	22(47.8)	20(43.5)	0.17(1)	0.67
**Relationship with patient**				
Sibling	40(87)	44(95.6)	1.16(2)	0.56
offspring	6(13)	2(4.4)		
**Current marital status**				
Married	13(28.3)	15(32.6)	1.15(2)	0.56
Single/Divorced	33(71.7)	31(67.4)		
**Current work situation**				
Fulltime	11(23.9)	11(23.9)	10.10(6)	0.12
Part-time	3(6.5)	12(26.1)		
Homemaker	13(28.3)	12(26.1)		
Student	6(13)	6(13)		
Retired	3(6.5)	0(0)		
Unemployed	6(13)	3(6.5)		
Other	4(8.7)	2(4.3)		
**Education**				
Illiterate	0(0)	2(4.4)	8.9(5)	0.11
Primary	5(10.9)	0(0)		
Elementary	17(37)	12(26.7)		
Diploma	10(21.7)	14(31.1)		
BS degree	5(10.9)	7(15.6)		
MS and higher	9(19.6)	10(22.2)		
**Family history of psychiatric**				
**Disorder (other than the proband)**				
None				
First degree	29(65.9)	28(63.7)		
Second degree	7(15.9)	10(22.7)		
Family history of psychiatric	8(18.2)	6(13.6)	0.83(3)	0.84

**Table 3:** Comparison of the Baseline Severity of Prodromal Symptoms between ‎the Groups with High- and Low-Risk for Psychosis

**Clinical Variables**	**High risk (N=46)**	**Low risk (N=46)**	**Man Whitney** **P value**
SIPS positive symptoms, mean (SD) Unusual thought content Suspiciousness Grandiose ideas Perceptual abnormalities Disorganized communication	3.03(1.64) 2.36(1.85) 0.71(1.33) 1.54(1.77) 0.29(0.66)	0.5 (0.64) 0.42(0.64) 0.12(0.33) 0.48(1.5) 0.02(0.16)	< 0.001* < 0.001* 0.05 < 0.001* 0.03
SIPS negative symptoms, mean (SD) Social anhedonia or withdrawal Avolition Decreased expression of emotion Decreased experience of self Decreased ideational richness Deterioration in role functioning	1(1.36) 1.29(1.61) 0.50(0.88) 0.50(1.04) 0.32(0.77) 1.57(1.71)	0.12(0.40) 0.22(0.58) 0.05(0.22) 0.12(0.40) 0.0 (0.0) 0.40(0.74)	< 0.001* < 0.001* 0.04 0.07 0.02* < 0.001*
SIPS disorganized symptom, mean(SD) Odd behavior or appearance Bizarre thinking Trouble with focus and attention Personal hygiene/ social attentiveness	0.11 (0.42) 0.36 (0.83) 1.82(1.89) 0.54(1.10)	0.0 (0.0) 0.0 (0.0) 0.65(1) 0.10(0.39)	0.12 < 0.001* < 0.001* 0.04
SIPS general symptoms, mean (SD) Sleep disturbance Dysphoric mood Motor disturbance Impaired tolerance to normal stress	1.71(1.70) 2.93(1.78) 0.0 (0.0) 2.21(1.77)	0.47(0.93) 1.10(1.08) 0.0 (0.0) 0.72(0.96)	< 0.001* < 0.001* 0.47 < 0.001*

**Tablel 4:** Comparison of the Follow-up Severity of Prodromal Symptoms‎ between the Groups with High- and Low-Risk for Psychosis

**Clinical Variables**	**High risk (N=46)**	**Low risk (N=46)**	**Man Whitney** **P value**
SIPS positive symptoms, mean (SD) Unusual thought content Suspiciousness Grandiose ideas Perceptual abnormalities Disorganized communication	2.43 (1.1) 1.82 (1.33) 0.61 (1.06) 1.07 (1.25) 0.29 (0.66)	0.45 (0.55) 0.35 (0.53) 0.12 (0.33) 0.25 (0.49) 0.02 (0.16)	< 0.001* < 0.001* 0.05 < 0.001* 0.03
SIPS negative symptoms, mean (SD) Social anhedonia or withdrawal Avolition Decreased expression of emotion Decreased experience of self Decreased ideational richness Deterioration in role functioning	0.75 (0.27) 0.71 (0.90) 0.32 (0.67) 0.46 (1.04) 0.25 (0.59) 1 (1.12)	0.07 (0.27) 0.15 (0.43) 0.0(0.0) 0.02 (0.16) 0.0(0.0) 0.37 (0.70)	< 0.001* < 0.001* 0.01* < 0.001* 0.02* 0.02*
SIPS disorganized symptom, mean(SD) Odd behavior or appearance Bizarre thinking Trouble with focus and attention Personal hygiene/ social attentiveness	0.11 (0.42) 0.36 (0.83) 1.21 (1.2) 0.25 (0.52)	0.0 (0.0) 0.02 (0.16) 0.60 (0.87) 0.02 (0.16)	0.12 0.01* 0.01* 0.01*
SIPS general symptoms, mean (SD) Sleep disturbance Dysphoric mood Motor disturbance Impaired tolerance to normal stress	1.25 (1.29) 2.04 (1.32) 0.0 (0.0) 1.40 (1.10)	0.47 (0.82) 1.25 (1.06) 0.0 (0.0) 0.62 (0.84)	0.01 < 0.001* 0.48 < 0.001*

**Graph 1:**
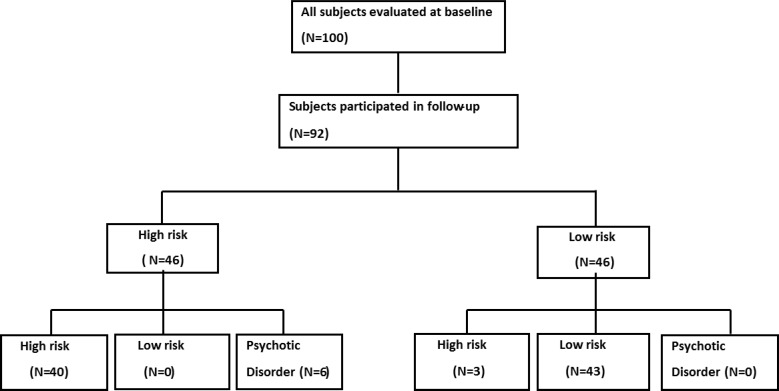
Flowchart of inclusion and follow-up of patient’s

***Reliability:***

At baseline, the Cronbach’s alpha coefficient for the SOPS total score was 0.89, with an alpha value of 0.69 for the positive ‎symptoms subscale, 0.81 for the negative symptoms subscale, 0.45 for the disorganization symptoms, and 0.69 for the general ‎symptoms subscale. Besides, at follow-up, the Cronbach’s alpha coefficient for the SOPS total score was 0.929, with an alpha level of ‎‎0.78, 0.78, 0.5 and 0.8 for the positive, negative, disorganization and general symptoms subscales, respectively.‎

***Validity:***

In order to assess the criterion validity of the SOPS scale, the total scores of SOPS were correlated with GAF scores. At baseline ‎evaluations, there was a significant indirect correlation between the SOPS total score and GAF scores with correlation coefficient of -‎‎0.7(P <0.001). Likewise at follow-up, there was a more significant and indirect correlation between the SOPS total score and GAF ‎score with correlation coefficient of -0.87(P <0.001).‎

High-risk group demonstrated significantly higher severity in all SIPS items at baseline, except in “odd behavior or appearance” and ‎‎“motor disturbance” in which the differences were not statistically significant with P values of 0.12 and 0.48, respectively (Table 3). After the 12-month follow-up, the high-risk group continued to illustrate higher severity in almost all SIPS items, which were ‎statistically significant. Interestingly, the difference in “odd behavior or appearance” and “motor disturbance” remained statistically ‎insignificant with p values of 0.12 and 0.47, respectively Table 4. Furthermore, the difference between the two groups appeared ‎statistically insignificant (P = 0.07) in “decreased experience of self” item at follow-up. As illustrated in Graph 1, six participants in ‎the high-risk group developed a full psychosis after a 12- month follow-up, which resulted in conversion rate of 13% (95% CI [0.029, ‎‎0.23]) and other participants remained high-risk through the follow-up period. Besides, three participants in the low-risk group ‎became high-risk -prodromal for psychosis- at follow-ups. None of the participants in the low-risk group converted to a full psychosis ‎in this period.‎

## Discussion

We found higher transition rate to full psychosis among the high-risk group compared to low-risk within one-year follow-up as we ‎demonstrated 13% transition rate to full psychosis in the high-risk group and zero in the low-risk participants. The high-risk group ‎had a significantly more severe positive, negative, disorganization, and general symptoms at baseline and continued to have more ‎severe symptoms at one-year follow-up. ‎

Previous studies have revealed a wide range of transition rates to full psychosis among people with prodromal syndrome during ‎different follow-up periods. For example, a study with 9.6 years follow-up reported 70% conversion rate (28). While Cannon and ‎colleagues reported conversion rates in 6, 12, 18, 24 and 30 months follow-ups point by point ( 12.7, 21.7, 26.8, 32.6, 35.3%), ‎respectively (30). In another study, conversion rate after the one- year follow-up were reported at 22%. [18] At first glance, it seems ‎that literature has reported higher rates of transition to full psychosis compared to our study. However, such a difference is probably ‎due to larger sample sizes, longer duration of follow-up or inclusion of persons with more severe symptoms. ‎

Furthermore, there is evidence for the application of prodromal syndromes for other mental disorders. In a recent study, attenuated ‎positive symptoms based on the SIPS has been shown to be associated with greater suicidality and psychopathology severity in a ‎sample of 13 to 35 year-old participants seeking mental help (37). Besides, considering the results of a 5-year prospective study of ‎adolescents with severe behavioral problems, the SIPS demonstrated limited power for anticipating psychosis, whereas it appeared ‎to be useful for mood and conduct disorders (38). These findings could propose the implementation of the SIPS not only as a ‎psychosis risk-screening tool, but also as a measure, assessing more global aspects of mental health.‎

A study conducted by Schlosser's et al. with 40 high-risk participants and one-year follow-up duration is similar to ours. They also ‎reported 12.5% transition rate to full psychosis, which is similar to our results. (36). Taken together, all of these investigations ‎reported a transition rates to psychosis between 6.6% (26) and 54% (27) with mean follow-up durations ranging between 6 to 12 ‎months [9]. We included patients with less severe prodromal symptoms in the high-risk group with relatively shorter duration of ‎follow-up; therefore, relatively low conversion rate to psychosis could be the result of our recruitment criteria. ‎

In contrast to our results, Schlosser showed that the severity of symptoms decreased in 36% of the ”clinical high risk” participants ‎during two-and-a-half year follow-up; and consequently, 30% of them experienced functional improvements. However, we ‎concluded that no one experienced a decline in severity of the symptoms or improvement in the total functional state. This could be ‎explained by larger sample size and longer duration of follow-up of Schlosser's study (30). In addition, we included persons with ‎minimum of 2 instead of 3 on the basis of attenuated psychotic syndrome which can explain higher proportion of the high-risk group ‎after a one- year follow-up in our study. ‎

## Limitations

This study had weaknesses that should raise caution in any interpretation of the findings: First, small sample size led to limitation in ‎detection of statistically significant differences and low conversion rates. Second was the short duration of the follow-up which may ‎have resulted in less precise transition rate to full psychosis. Third, validity of some SIPS items for Persian speaking patients is in ‎question; e.g., some items that assess “unusual thought content” and “perceptual abnormalities” were vague and hardly ‎understandable for some participants. Further validation studies of the Persian translation are warranted. Fourth, we did not use a ‎comprehensive assessment tool to confirm diagnosis at the end of the follow-up period. The assessments would have been improved ‎if we had confirmed our diagnosis after the one-year follow-up with structured instruments such as the Structured Clinical Interview ‎for DSM-IV (SCID). However, we were more interested in detecting transition to any psychotic illness rather than any particular ‎disorder. Nevertheless, this study has some strengths including low rate of loss to follow-up, having a control group, being the first ‎study using the Persian translation of the SIPS and measuring transition to full psychosis rate of at-risk subjects among relatives of ‎patients with schizophrenia.‎

## Conclusion

This study confirmed the predictive validity of prodromal syndromes for future psychosis in high-risk participants. In a group of first-‎degree relatives of patients with schizophrenia, the presence of prodromal syndromes not only raises the risk of conversion to ‎psychosis, but also prodromal psychotic symptoms appeared to be persistent at least for one year.‎
